# Clinical evaluation of resin infiltration treatment masking effect on hypomineralised enamel surfaces

**DOI:** 10.1186/s12903-023-03140-6

**Published:** 2023-07-03

**Authors:** Halenur Altan, Rabia Erağca Yilmaz

**Affiliations:** 1 Department of Pediatric Dentistry, Faculty of Dentistry, Necmettin Erbakan University, Konya, Turkey; 2 Department of Pediatric Dentistry, Dental Oral and Dental Health Polyclinic, Tokat, Turkey

**Keywords:** Molar incisor hypomineralization, Resin infiltration, Enamel, Lesion, Cross polarisation, Laser fluorescence

## Abstract

**Background:**

Resin infiltration is a micro-invasive treatment for molar incisor hypomineralisation (MIH). In this study it was aimed to evaluate the masking effect of resin infiltration treatment (ICON) on hypomineralised enamel surface of permanent anterior teeth by using laser fluorescence, spectrophotometer, and cross-polarisation photography.

**Methods:**

A total of 116 permanent central incisors in 37 patients were included in the study. The resin infiltration treatment (Icon®) was applied to the teeth with MIH; the healthy teeth received no treatment (control). Hypomineralised enamel lesions were evaluated by ICDAS II criteria. DIAGNOdent Pen was used to assess the lesions and healthy enamel surface quantitatively. Colour changes in enamel lesions were evaluated by using a spectrophotometer (VITA EasyShare). Each enamel lesion was imaged using a cross-polarization technique before and after treatment. All photos were assessed using Image J to evaluate the changes in lesion size. Enamel lesions were evaluated before; immediately after; 1; 3; and 6 months after treatment. Statistical significance was set as *p* < 0.05.

**Results:**

After the resin infiltration, significant decreases were found in the mean DIAGNOdent values for the treatment group (*p* < 0.05). The colour differences before and after treatment significantly differed in all follow-ups (*p* < 0.05). In the treatment group, lesion areas decreased significantly after treatment (*p* < 0.05).

**Conclusions:**

The resin infiltration treatment has a masking effect on MIH lesions without cavities, with stable outcomes after six months. The cross-polarization photography technique may be use to evaluate the lesion size instead of photography with flash.

Trial registration.

NCT04685889 (registered 28 December 2020).

## Background

One of the developmental deficiencies of the enamel is clinically visible enamel opacity. These deficiencies can arise from different types of anomalies (e.g. molar incisor hypomineralisation (MIH), amelogenesis imperfecta, fluorosis and consequences of trauma to the primary teeth) [1]. MIH is a clinical manifestation of enamel hypomineralisation in which permanent incisors and one or more first permanent molars may be affected [2, 3]. It occurs when ameloblasts damaged during the maturation stage are not able to secrete the organic matrix appropriately [3, 4]. While the primary etiology of MIH is unknown, theoretically, it is indicated that, changes in the matrix pH during enamel maturation may lead to MIH. The potential aetiological factors are systemic diseases, antibiotic use, preterm delivery, environmental toxins, and malnutrition [1, 5].

Dentine sensitivity, post-eruptive enamel breakdown, pulpal inflammation, dental pain, and early tooth extraction are frequently seen in teeth affected by MIH [6, 7]. Further, MIH alters the tooth’s aesthetic features, such as opacity, translucency, and fluorescence [8]. The clinical characteristics of MIH are well-demarcated hypomineralised borders, porous defects and altered enamel translucency. These changes affect enamel colour which ranges from white cream to yellow brown, that allow clinicians to separate the adjacent normal enamel from the coloured enamel. The defective enamel has a smooth surface with average thickness; however, the enamel structure is defective. As a characteristic of MIH is that the cervical third is spared, but the two other thirds might be affected [9].

Resin infiltration treatment has been proposed recently as an alternative micro-invasive option for enamel defects [10]. In caries ethology, main purpose of the resin infiltration treatment, using resin sealing of the microporosities which causes diffusion pathways for acids and dissolved minerals, thus arresting the incipient caries lesion. The resin matrix can also strengthen the enamel structure mechanically. Removal of the surface layer might also weaken the lesion structure, but Meyer-Lueckel et al. [11] reported that 15% HCl is most suitable for removing approximately 40 μm of the hypermineralized surface layer. Moreover, in this study, no cavitation occurred after etching, even when the surface layer had been eroded completely. In practice, subsequent resin infiltration should ensure restrengthening of the lesion structure. In contrast, microabrasion, which has been commonly used in white spots, removes up to 360 μm of enamel [12]. 99% ethanol is used to remove the water stored inside the lesion body's microporosity and allow the resin to penetrate into the lesion body driven by capillary forces [13].

Enamel lesions can be examined by various methods other than clinical examination [14]. One such is laser fluorescence (DIAGNOdent Pen), which utilizes the laser**s** fluorescence diagnostic method [15]. The light absorbed by the teeth is reflected in the form of fluorescence. The fluorescent light's intensity increases with the lesion's increasing depth. Another method is cross-polarisation, which is more reliable than traditional intraoral photography as it enhances the appearance of enamel defects by reducing the reflection on the enamel surface [16]. The enamel's structural details on the tooth's anterior surface may not be visible in the clinic due to light reflection. Direct light reflection from the anterior surface of the teeth may cause loss of data enamel defects may combine or wholely remove degree of probable enamel defects [16].

As the resin infiltration protocol was developed for managing carious lesions, the application for treating hypomineralized lesions should be developed. In this study, it was aimed to quantitatively evaluate the masking effect of resin infiltration treatment (ICON) on hypomineralized enamel surface lesions by using DIAGNOdent Pen; changes in colour by using a spectrophotometer, changes in lesion size by using cross-polarisation photography in permanent incisor teeth with MIH after before treatment; immediately after treatment; 1; 3; and 6-month follow-up.

## Methods

The study was approved by the Tokat Gaziosmanpaşa University Clinical Research Local Ethics Committee (Project no: 18-KAEK-094), and the protocol was registered at Clinical Trials.gov (NCT04685889). The data were presented in accordance with the CONSORT statement. The written informed consent form was obtained from parents of the children included in the study stating that they accepted the treatment. This study was performed in accordance with the ethical standards of the Declaration of Helsinki (1964) and its subsequent amendments.

### Participants selection

This non-randomized, controlled clinical study was developed at the pediatric dentistry clinic in Tokat Gaziosmanpaşa University, Faculty of Dentistry, Tokat, Turkey. A total of 37 patients (21 girls; 16 boys) aged between 8 and 14 years, with MIH on the labial surface of their permanent central incisors, were included in this study. The inclusion criterion was: labial surface of at least one permanent anterior central tooth affected by MIH. The exclusion criteria included: dental caries and filling, dental anomaly on anterior teeth, periodontal disease**s**, undergoing orthodontic treatment, and cognitive and behavioral conditions.

### Sample size of the study

**Fig. 1:**
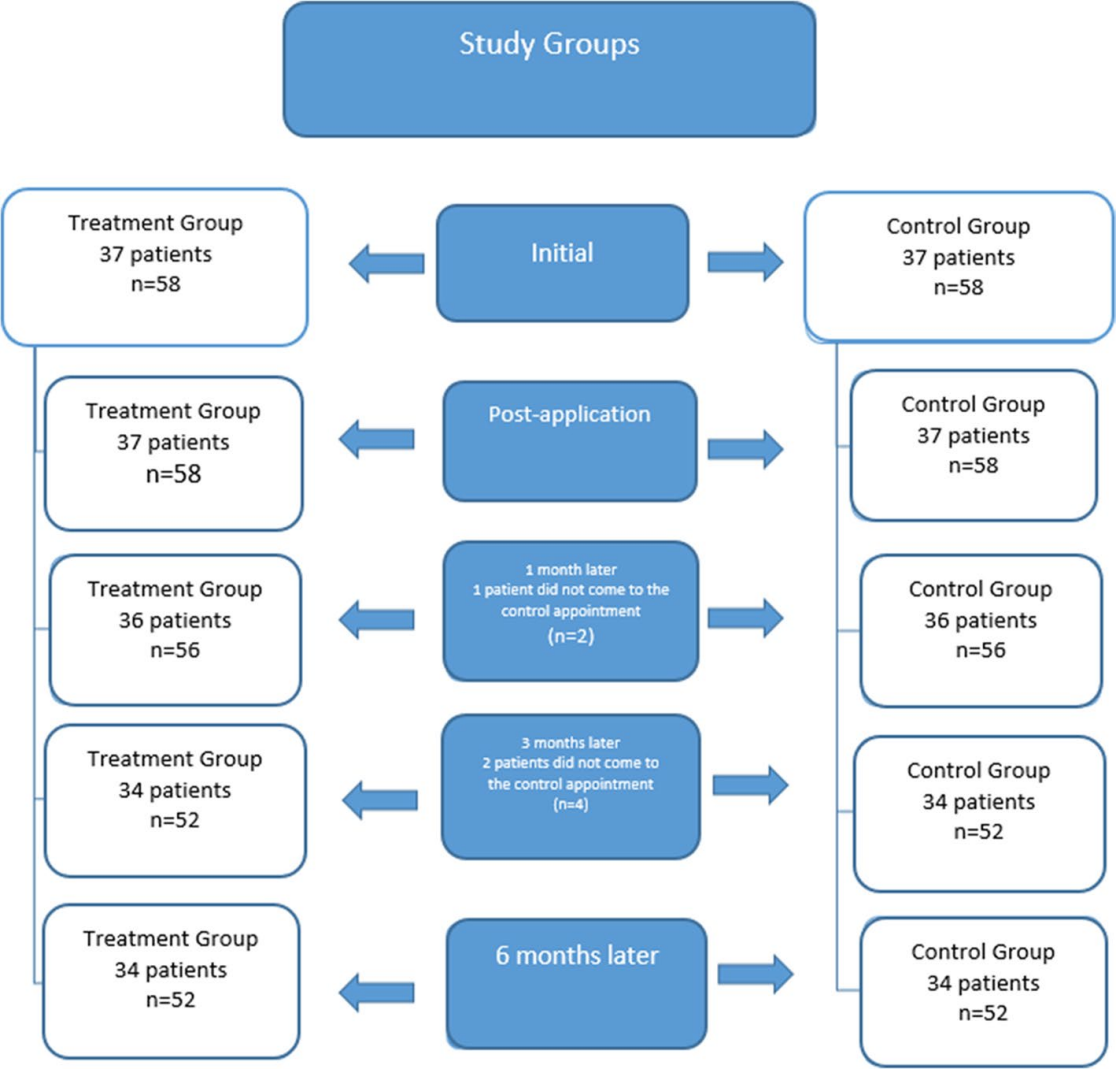
Flow-chart of the study design

The sample size was calculated at a significance level of 0.05 with statistical power of 90% and an effect size of 0.307. The analysis was performed using the G*Powers software program (version 3.1.9.6), which required a sample size of 96 teeth. In order to account for possible exclusions, the sample size was increased to 116 teeth. Three patients who did not attend follow-up appointments were excluded from the study (Fig. 1).

### Interventions

Group I (Treatment Group): Icon® resin infiltration treatment was performed on 58 permanent central incisors with MIH, which were evaluated before; immediately after; and 1, 3, and 6 months after the procedure.

Group II (Control Group): No treatment was performed on the healthy teeth. However, like the treated teeth, 58 healthy permanent central incisors in the same individuals were evaluated before, immediately after, and 1, 3, and 6 months after the procedure.

### Resin infiltration process

The affected teeth were isolated using a rubber dam (Royal Shield Powder Free Latex Dental Dams, Malaysia) and a clamp (YDM, Tokyo, Japan) before the application of Icon®. A sufficient amount of %15 hydrochloric acid was applied to the lesion using the Icon® etch syringe included in the set and was allowed to sit for 2 min. After washing it with water for 30 s, the tooth was dried using an air spray. Once the enamel surface appeared chalky, Icon® dry application was initiated.

**Fig. 2:**
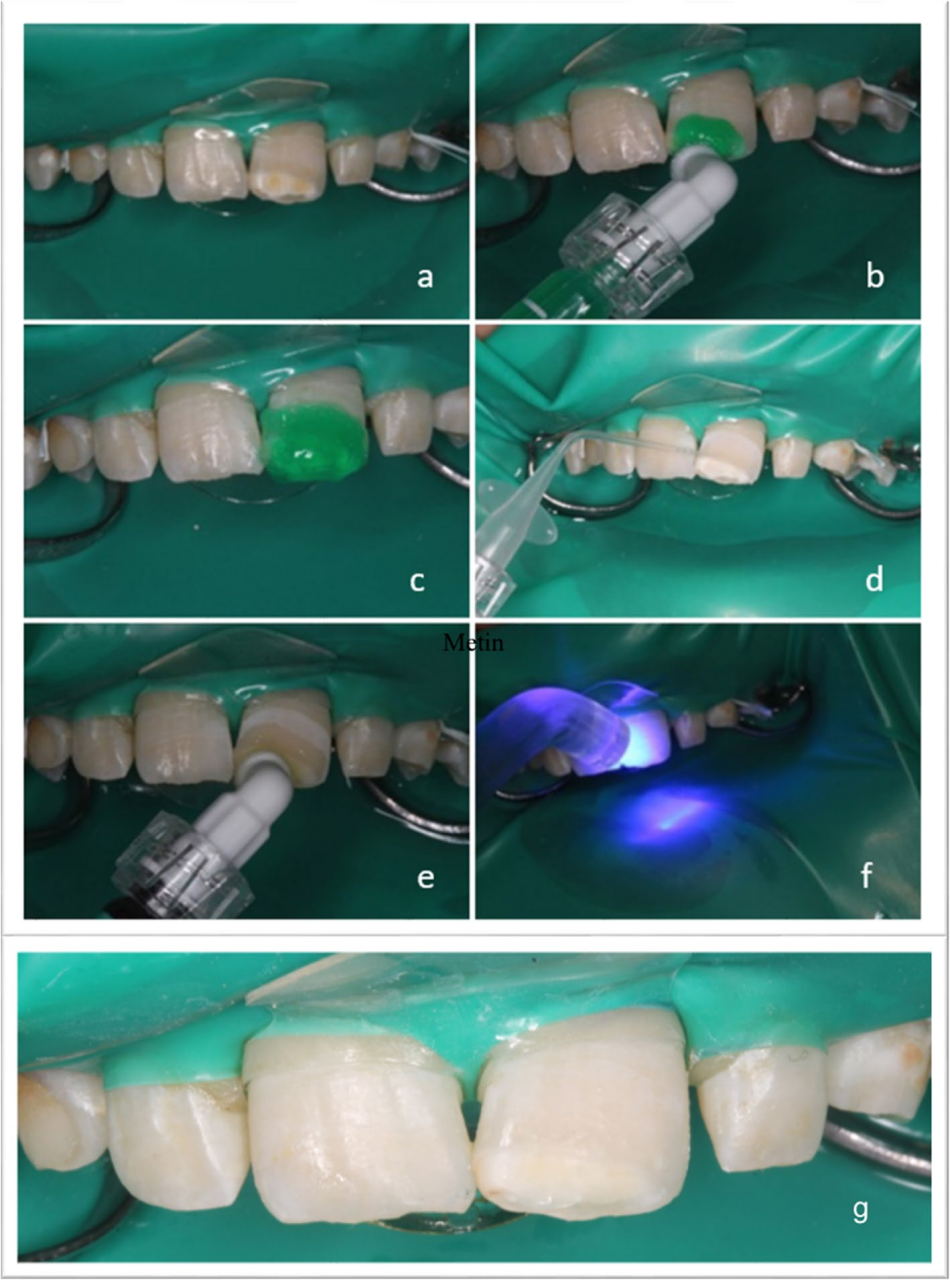
Steps in Icon® application (**a**: Isolation with rubber dam; **b**: Acid application with etch syringe; **c**: Form of the tooth after acid application; **d**: Ethanol application; **e**: Application of resin; **f**: Polymerisation with light); **g**: Clinic photography after post treatment

Ethanol was applied to the lesion using the Icon® dry syringe included in the set, and after 30 s, it was dried using an air spray. A sufficient amount of resin infiltrant was applied to the lesion using the Icon® infiltrant syringe. The resin infiltrant was applied to the lesion surface by massaging the tip of the syringe on the lesion in circular movements for 3 min, such that it penetrated the surface thoroughly. The resin infiltrant was polymerized for 40 s with a light-cure device (3 M ESPE Elipar S10, U.S.A.). Subsequently, the material was applied for the second time. One minute was provided for the material to infiltrate the pores, after which it was polymerized for 40 s. When the procedure was complete, the rubber dam and clamps were removed, and surface finishing was performed using fine-grained polishing burs and rubbers (Fig. 2).

### Quantitative evaluation of enamel lesions using the DIAGNOdent Pen

To prevent false positive results in the measurements performed with the DIAGNOdent Pen (Kavo, Biberach, Germany), plaque on the teeth was removed by polishing, and the teeth were air-dried for 5 s. Sapphire tip number 2 was used for the measurements performed with the DIAGNOdent Pen. The measurement was repeated at three different parts of the hypomineralised surface, and the highest value was recorded as the laser fluorescence value. These measurements were performed before; immediately after; and 1, 3, and 6 months after the procedure, in both the treatment and control groups.

### Colour determination using a spectrophotometer

Changes in tooth colour of the treatment and control groups were measured using the VITA EasyShare Advance® (Vita Zahnfakrik, Germany) device. Before measurement, the teeth were isolated and air-dried for 5 s. The measurements were repeated three times for each tooth, and the averages of the L*, a*, and b* values of the International Commission on Illumination (CIE) scale were recorded. The changes in tooth colour were measured before; immediately after; and 1, 3, and 6 months after resin application in both the treatment and control groups. The teeth' L*, a*, and b* values and the subsequently derived ΔE values were recorded.

### Obtaining intraoral photos using cross-polarisation photography

**Fig. 3:**
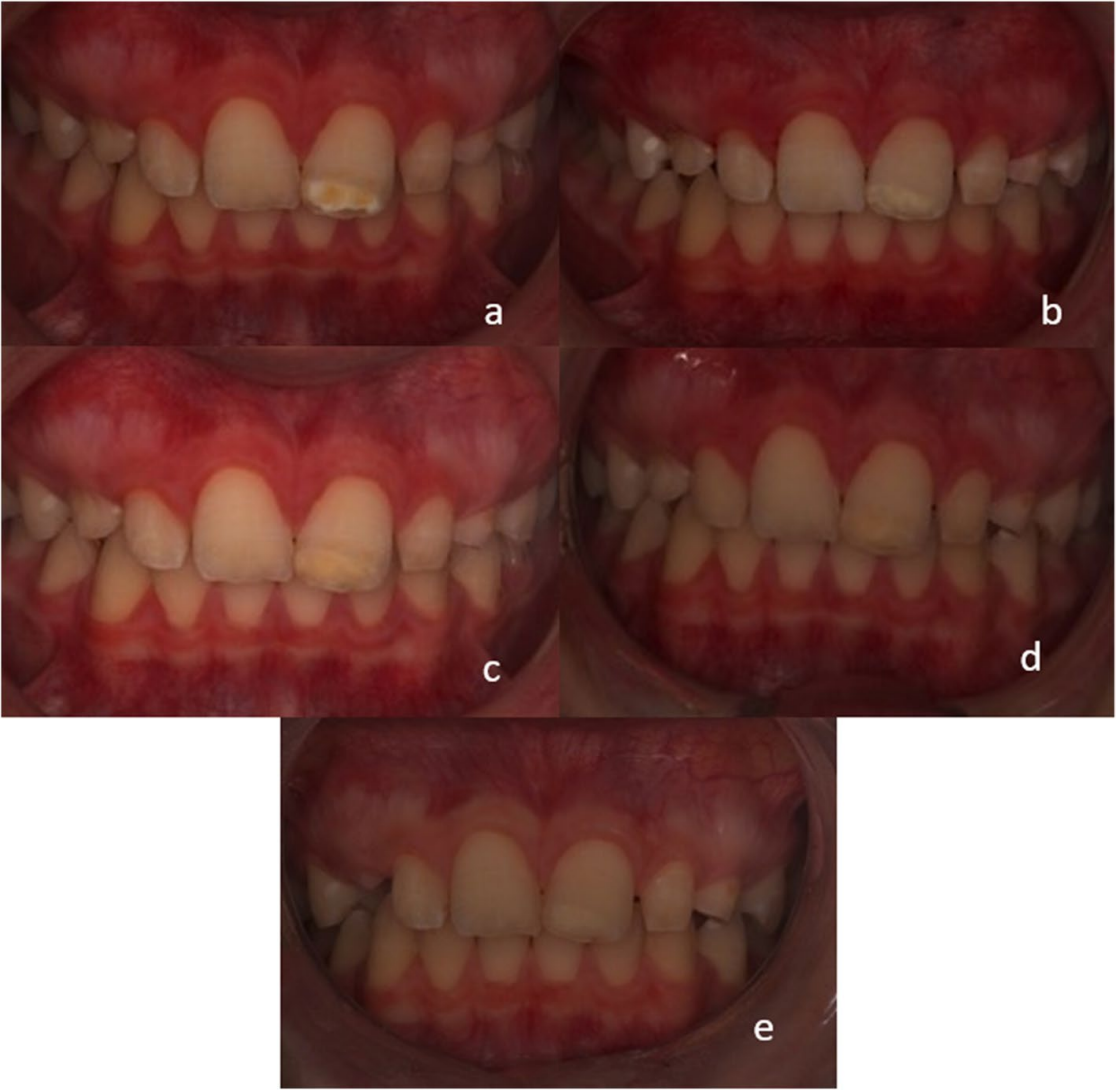
Intraoral photographs of a patient with MIH lesion in tooth #21 obtained using cross-polarization photography (**a**: Before treatment; **b**: Immediately after Icon® application; **c**: 1-month control; **d**: 3-month control; **e**: 6-month control)

A cross-polarisation filter suitable for our ring flash (Yongnuo YN-14EX TTL, China) was prepared and attached to the camera (Canon EOS 700D, Tokyo-Japan) before obtaining the intraoral photographs in order to minimize light reflection and to be able to observe the lesion borders more clearly. After the teeth were removed using a photo retractor, intraoral photographs of the treatment and control groups were obtained before; immediately after; and 1, 3, and 6 months after the application of the resin and were archived. Next, these images were analyzed using Image J (1.31o, National Institutes of Health, Bethesda, MD, USA) software, and the lesion areas of the teeth in the treatment group were measured in mm^2^. The measurements were repeated twice at different times. Since these repeated measurements were found to agree with each other, their averages were taken as the final result (Fig. 3).

### Statistical analysis

The data were analyzed using IBM Statistical Package for the Social Sciences v23. Compliance with the normal distribution was examined using the Kolmogorov-Smirnov test. The independent t-test was performed to compare two normally distributed quantitative variables, and the Mann-Whitney U test was used for non-normally distributed data. One-way analysis of variance was used for the comparison of normally distributed quantitative data from more than two groups. In contrast, the Kruskal-Wallis test was used to compare non-normally distributed quantitative data. A paired two-sample t-test was used to compare two normally distributed timepoint datasets, and the Wilcoxon test was used to compare non-normally distributed data. Data were considered significant when p < 0.05.

## Results

A total of 116 permanent central incisors in a set of 37 patients, including 58 teeth with developmental enamel defects due to MIH and 58 healthy teeth, were evaluated in this study. The patients included 21 girls (mean age = 9.94 ± 2.04) and 16 boys (mean age = 9.43 ± 2.08) in the 8–14 years (9.70 ± 2.08) age group.

### Quantitative evaluation of enamel lesions using the DIAGNOdent Pen

The averages Diagnodent values in treatment and control groups and their statistical comparisons are presented in Table 1.

**Table 1:** Comparison of DIAGNOdent Pen values of the treatment and control groups

	Treatment group	Control group
DIAGNOdent Pen value before treatment	21.96 ± 15^a,A^	2.6 ± 0.57^a,B^
DIAGNOdent Pen value immediately after treatment	12.98 ± 9.95^b,A^	2.6 ± 0.57^a,B^
1 month control DIAGNOdent Pen value	13.44 ± 9.69^b,A^	2.79 ± 0.82^b,B^
3 months control DIAGNOdent Pen value	13.12 ± 9.82^b,A^	2.6 ± 0.57^a,B^
6 months control DIAGNOdent Pen value	13.4 ± 9.88^b,A^	2.81 ± 0.5^b,B^

The paired two-sample t-test and independent t-test were used. Lower case letters indicate in-group evaluation between rows; capital letters indicate evaluation between the columns. The same letters indicate that there is no statistically significant difference (*p* > 0.05), and different letters indicate that there is a statistically significant difference (*p* < 0.05)

In the treatment group, values measured initially with the laser fluorescence (DIAGNOdent Pen) were significantly higher compared to those measured immediately after resin application and in the 1^st^, 3^rd^, and 6^th^ months after application (*p* < 0.05). In the control group, there was no statistically significant difference between the values measured before the application versus those measured immediately after and three months later (*p* > 0.05). However, the values measured after 1 and 6 months were significantly higher than the initial values (*p* < 0.05). There was a statistically significant difference between the values of the treatment and control groups at all times (*p* < 0.05).

### Analysis of changes in colour of affected teeth as measured by a spectrophotometer

#### Changes in L* Value

The L* value is a parameter that indicates the brightness of the colour. In the treatment group, no change in the L* values measured immediately after treatment compared to the initial values (*p* > 0.05). However, the L* values measured at months 1, 3, and 6 were significantly lower than the initial values (*p* < 0.05). While there was no significant difference in the L* values of the control group immediately after treatment and 1 month later compared to the initial values (*p* > 0.05), there was a significant decrease in the values after 3 and 6 months (*p* < 0.05). Moreover, there was a darkening of the tooth colour as well. There was a significant difference between the L* values of the treatment and control groups at all times (*p* < 0.05).

#### Comparison of ΔE Values

The average ∆E values of the teeth in the treatment and control groups and their statistical comparisons are presented in Table 2.In the treatment group, no significant difference was found between the ∆E values calculated immediately after the treatment, and 1, 3, and 6 months after the treatment, compared to the initial values (*p* > 0.05). In the control group, there was no significant difference between the ∆E values calculated immediately after the treatment, and 1, 3, and 6 months after the treatment, compared to the initial values (*p* > 0.05). When the ∆E values of the treatment and control groups were compared, there was a statistically significant difference at all times (*p* < 0.05).

**Table 2:** Comparison of ∆E values of the treatment and control groups

	Treatment group	Control group
∆E1a	9.99 ± 6.98^a,A^	5.43 ± 5.78^b,B^
∆E2a	11.3 ± 8.99^a,A^	3.75 ± 4.77^b,B^
∆E3a	11.12 ± 7.66^a,A^	4.94 ± 5.56^b,B^
∆E4a	11.54 ± 7.13^a,A^	4.53 ± 5.59^b,B^

The Kruskal–Wallis test statistic and independent t-test were used. Lower case letters indicate in-group evaluation between rows; capital letters indicate evaluation between the columns. The same letters indicate that there is no statistically significant difference (*p* > 0.05), and different letters indicate that there is a statistically significant difference (*p* < 0.05)

∆E1a: Colour difference between before treatment and immediately after treatment

∆E2a: Colour difference between before treatment and 1 month after treatment

∆E3a: Colour difference between before treatment and 3 months after treatment

∆E4a: Colour difference between before treatment and 6 months after treatment

### Evaluation of lesion areas by using cross-polarisation photography

Since the measurement of the lesion areas was relative, two measurements were performed at different times. Intraclass correlation(ICC=0.996-0.988) indicates excellent high reliability (0.9 range to 1). The average values of the lesion areas(mm^2^) and their statistical comparisons are presented in Table 3.

**Table 3:** Comparison of lesion areas

	Treatment Group (mm^2^)
Lesion area before treatment	4.9 ± 3^a^
Lesion area immediately after treatment	4.6 ± 3^b^
1-month control lesion area	4.7 ± 3,1^b^
3-month control lesion area	4.7 ± 3,1^b^
6-month control lesion area	4.7 ± 3,1^b^

The paired two-sample t-test was used. The same letters indicate that there is no statistically significant difference (*p* > 0.05); different letters indicate that there is a statistically significant difference (*p* < 0.05)

In the treatment group, the lesion areas measured immediately after treatment, and 1, 3, and 6 months later were significantly smaller than the initial values (p < 0.05). There was no significant difference between the lesion areas immediately after treatment and 1, 3, and 6 months later (p > 0.05). After the Icon® application, there was a decrease in the lesion area, which remained stable for 6 months.

## Discussion

The use of infiltrants for masking enamel discolourations in hypomineralized teeth has spread more recently [9]. We evaluated the qualitative and quantitative clinical outcomes of resin infiltration on teeth with MIH for 6 months. Our results showed that resin infiltration using Icon® was a successful effect in masking of hipomineralised teeth in children with MIH.

The primary mechanism of DIAGNOdent is based on different absorption and scattering of laser fluorescence radiation of the carious lesion compared to the surrounding healthy tissue [15]. Changes caused by caries in tooth cause an increase in fluorescence at the exciting wavelength. It was reported that this high fluorescence is due to bacterial porphyrins in the carious lesion [15, 17]. In MIH, opacities structure differs from caries etiology [9, 18]. DIAGNOdent readings were recorded high in tooth with irregular enamel structure without dental caries as MIH [19]. One of the reasons for higher values in the non-carious but hypomineralised enamel tissue might be the increased protein in the enamel [20]. Another reason, the fluorescence of darker teeth due to intrinsic enamel stains is higher than whiter teeth [15]. In line with previous studies, the values obtained using the laser fluorescence (DIAGNOdent Pen) were higher in MIH (white-cream and yellow–brown localized opacities without cavitation) before the application of resin infiltrant compared to the healthy anterior teeth.

Histological studies have shown an increase in microporosity within the different layers of hypomineralised enamel lesions [2, 18]. The porous and enlarged intercrystalline spaces act as diffusion pathways for acids and dissolved minerals. The application of a low-viscosity resin infiltrant fills the pores and reduces the microporous structure. Assuming the resin does not penetrate the sublayers of the enamel lesion and is only limited to the outermost layer. In that case, the effectiveness of the resin infiltration treatment might be decreasing over time [21]. In our study, the fact that the values obtained using the laser fluorescence (DIAGNOdent Pen) in the teeth treated with resin infiltrant decreased and, after that, remained stable for 6 months might suggest that the resin was homogeneously infiltrated into the lesion.

Nowadays, many colour systems are used in restorative and prosthetic dental treatments; the most preferred system in recent years is the CIELAB (ΔEab) colour system [22]. The L*, a*, and b* numerical values in the CIE scale in both the treatment and control groups were determined by VITAEasy Shade Advance®, and the ΔE numerical values depicting the change between colours were also calculated. Although the ΔE value refers to the colour change in the tooth, it does not provide information about the direction of colour change. Therefore, ΔE and L* values were evaluated together in our study. An increase in the L* value of teeth with enamel lesions is considered an increase in the brightness of the lesion area, representing greater lesion depth. A decrease in the L* value is considered a masking of the lesion, i.e., darker-coloured lesion areas resembling healthy enamel [23, 24]. In our study, a significant decrease was observed in the L* value after 6 months, which implied that the colour of the hypomineralised lesions had darkened and approached the natural tooth colour.

In this study, we found that the ΔE values calculated immediately after resin infiltration treatment and those calculated 1, 3, and 6 months later were, on average, greater by a factor of ΔE 3.7 compared to the initial values. Moreover, the colour change was clinically perceptible after resin infiltration treatment, as reported in the literature. However, the colour change was less in the control group compared to that in the treatment group at all times [25, 26]. Further, when the resulting colour change was evaluated with the L* values, it was determined that the colour of the lesions had darkened. The discolouration observed before and immediately after resin infiltration treatment in the control group may have been caused due to isolation of the teeth from oral fluids and dehydration for approximately 30 minutes during the application of the material and the measurements being performed.

The length of etching and drying application time affects the homogeneity and depth of the resin infiltration. C. Kobbe, et al.[27] proposed that two or three etching to obtain better aesthetic results in resin infiltration in white spot lesions. Hypomineralized enamel teeth has high organic content as protein and this may be a reason for the more difficult penetration of the infiltrant [27]. In this study, the etching and drying application was applied one time. If these processes were applied more time, maybe higher success could be achieved.

In their study, Khanna et al.[28] investigated the masking effectiveness of resin infiltration. Photographs of 70 teeth with opaque enamel lesions were obtained before and after treatment, and L*, a*, and b* values of these teeth were obtained using the Image J software. Unlike the aforementioned study, the colour parameters in our study were determined directly using VITA EasyShare Advance®, thereby minimizing errors caused by reflection during the photo shoot. Image J was not used to analyze the colour but to measure and compare the areas of hypomineralised lesions in the photographs obtained by cross-polarisation photography.

Similar to the aforementioned studies, we observed a significant decrease in the lesion areas after resin infiltration treatment (Icon®), which remained stable for 6 months. Since measuring the lesion areas using Image J is an advanced and repeatable method, and millimetric measurements obtain accurate results, it may be preferred over measurements performed using a periodontal probe or the naked eye in the clinic. We consider that using cross-polarisation photography provides more accurate information about the severity of MIH compared to visual examination or regular photo shoots. Studies on this subject are rare, which warrants more studies to support this information.

## Conclusion

Resin infiltration treatment (Icon®), provided effective results as a micro-invasive treatment of anterior teeth with MIH lesions. The cross-polarization photography technique may be use to evaluate the lesion size instead of photography with flash. However, there is a need for more clinical studies evaluating the long-term success of this material.

## Data Availability

The datasets used and/or analyzed during the current study are available from the corresponding author on reasonable request.

## Abbreviations

MIH: Molar Incisor Hypomineralisation
pH: Potential of hydrogen
CIE: International Commission on Illumination
nm: Nanometer
RI: Refractive Index
ΔEab: CIELAB
SPSS: Statistical Package for the Social Science
